# Estimation of lung vital capacity before and after coronary artery bypass grafting surgery: a comparison of incentive spirometer and ventilometry

**DOI:** 10.1186/1749-8090-6-70

**Published:** 2011-05-12

**Authors:** Areli Cunha Pinheiro, Michelli Christina Magalhães Novais, Mansueto Gomes Neto, Marcus Vinicius Herbst Rodrigues, Erenaldo de Souza Rodrigues, Roque Aras, Vitor Oliveira Carvalho

**Affiliations:** 1 Faculdade Social, Salvador, Bahia, Brazil; 2 Instituto do Coração do Hospital das Clínicas da Faculdade de Medicina da USP (InCor HC-FMUSP), São Paulo, Brazil; 3 Hospital Ana Neri da Faculdade de Medicina da Universidade Federal da Bahia (HAN-UFBA), Salvador, Bahia, Brazil; 4 Faculdade de Medicina da Universidade Federal da Bahia, Salvador, Bahia, Brazil

**Keywords:** Assessment, Vital Capacity, Cardiac surgery, physiotherapy, exercise

## Abstract

**Background:**

Measurement of vital capacity (VC) by spirometry is the most widely used technique for lung function evaluation, however, this form of assessment is costly and further investigation of other reliable methods at lower cost is necessary. Objective: To analyze the correlation between direct vital capacity measured with ventilometer and with incentive inspirometer in patients in pre and post cardiac surgery.

**Methodology:**

Cross-sectional comparative study with patients undergoing cardiac surgery. Respiratory parameters were evaluated through the measurement of VC performed by ventilometer and inspirometer. To analyze data normality the Kolmogorov-Smirnov test was applied, for correlation the Pearson correlation coefficient was used and for comparison of variables in pre and post operative period Student's t test was adopted. We established a level of ignificance of 5%. Data was presented as an average, standard deviation and relative frequency when needed. The significance level was set at 5%.

**Results:**

We studied 52 patients undergoing cardiac surgery, 20 patients in preoperative with VC-ventilometer: 32.95 ± 11.4 ml/kg and VC-inspirometer: 28.9 ± 11 ml/Kg, r = 0.7 p < 0.001. In the post operatory, 32 patients were evaluated with VC-ventilometer: 28.27 ± 12.48 ml/kg and VC-inspirometer: 26.98 ± 11 ml/Kg, r = 0.95 p < 0.001. Presenting a very high correlation between the evaluation forms studied.

**Conclusion:**

There was a high correlation between DVC measures with ventilometer and incentive spirometer in pre and post CABG surgery. Despite this, arises the necessity of further studies to evaluate the repercussion of this method in lowering costs at hospitals.

## Background

Vital capacity (VC), defined as the maximum amount of air that can be exhaled after a maximum inhalation, is an indispensable measure for the diagnosis of pulmonary mechanical limitation as well as for adequation of pulmonary reexpansion therapy applied to patients after cardiac surgery. The normal value of the VC is from 65 to 75 ml/kg, however, there may be variations regarding ethnicity, age, gender, height and weight [1-5].

The evaluation of pulmonary volumes and capacities is essential to characterize pulmonary mechanical limitation, especially in postoperative cardiac surgery patients [6,7].

It has been described that VC lower than 25 ml/Kg can predispose atelectasis, hypoxemia and inefficient cough [6,7]. After cardiac surgery, the impairment of VC has a multifactorial meaning and the restrictive pattern can last for more than 116 days, predisposing atelectasis and post operatory complications [8-16]. In order to obtain pulmonary volumes and capacities, spirometry and ventilometry are the most used techniques in clinical practice, nevertheless, both methods are very costly and not always available in hospitals [3,5,17-20].

Due to its low cost, incentive spirometers are widely used in hospitals. They are used for treating and preventive purposes regarding pulmonary complications. This device works with visual stimulation to deep inspiration and is largely used by patients in post operatory periods of abdominal and thoracic surgery [21,22].

Due to the importance of accessing VC in patients who underwent cardiac surgery and considering the high cost of ventilometers and spirometries, arises the necessity of an inexpensive alternative method which can reflect in a reliable manner the VC.

The aim of this study was to evaluate the incentive spirometers as a method of assessment VC in patients in pre and post coronary artery bypass grafting (CABG) surgery.

## Methods

### Studied population

This study was accomplished in a tertiary cardiac hospital with a group of patients in pre operatory and another group in the 5^th ^post operatory day of CABG surgery. Patients with smoking history, pulmonary diseases, extracorporeal circulation time higher than 150 minutes, intolerance and/or difficulties in understanding the technique were excluded.

This protocol was approved by the Ethical Committee of our institution. All patients provided informed consent prior to participation.

### Study design

This cross-sectional prospective study was designed to study the direct vital capacity (DVC, ml/Kg) measured by ventilometer and Incentive spirometer before and after CABG surgery.

The execution order of DVC techniques measured with ventilometer and incentive spirometer were randomized through sealed envelopes. All participants were oriented regarding the methodology to be used in each measurement. In order to avoid bias in the results presented, data collection was made by only one researcher.

### DVC Measurement with ventilometer

In order to measure DVC with ventilometer, the individuals were placed in a sitting position with thorax in a vertical way in approximately 90°. A ventilometer (Ferraris^®^) was used with a trachea in its shortest length, connected between the ventilometer and a hard flat mouthpiece. A nasal clip was used to avoid air escape by the nose.^23 ^Then, the patients performed deep inspiration until total pulmonary capacity followed by continuous and slow expiration until residual volume. The technique was applied three times and the highest volume was considered.

### DVC Measurement with incentive inspirometer

To assess DVC with an incentive spirometer, patients adopted the same positioning performed by the ventilometer. An inspirometer (Coach^®^) was used was used with a trachea in its shortest length, connected between the ventilometer and a hard flat mouthpiece. A nasal clip was used to avoid air escape by the nose. Then the patients performed deep slow expiration until residual volume, followed by continuous and deep inspiration until total pulmonary capacity, in which VC was measured through the numerical marking of the inspirometer. The technique was applied three times and the highest volume was considered.

### Statistical analysis

Descriptive statistics was applied to analyze demographic and clinical data, continuous variable information were assessed as measures of central tendency and dispersion and expressed as averages and standard deviation. Dichotomous or categorical variables were evaluated with frequency measures and presented as percentages. To analyze data normality *Kolmogorov-Smirnov *test was applied. Since data was regularly distributed the correlation assessment between DVC measured with ventilometer and incentive inspirometer was based on *Pearson *correlation coefficient [23,25]. T-student test for independent samples was used to compare patients' variables in pre and post operatory periods. Bland-Altman plots with 95% limits of agreements were also derived. The assessment occurred with use of *software SPSS (Statistical Package for the Social Sciences) for Windows *(version 14.0).

## Results

Fifty two patients submitted to CABG surgery were evaluated, 20 patients in pre operatory (15 men) and 32 patients in post operatory (21 men) (table 1). In table 2 averages and DVC standard deviation patterns accomplished through ventilometry and spirometry in pre and post operatory are described. The use of incentive spirometer was well tolerated and of easy comprehension by the patients.

**Table 1:** Demographic characteristics of patients in pre and post operatory cardiac surgery

VARIABLES N = 52	PRE OPERATORY N (%) AVERAGE ± SD	POST OPERATORY N (%) AVERAGE ± SD
**Gender**		
**Male**	**15 (75%)**	**21 (65%)**
**Female**	**05 (25%)**	**11 (35%)**
**Age**	**49.7 ± 15.13 anos**	**49.7 ± 15.8 anos**
**BMI**	**24.8 ± 3.3 kg/m^2^**	**25.5 ± 2.5 kg/m^2^**
**RF**	**18.4 ± 6 rpm**	**21.2 ± 7.94 rpm**
**TV**	**717.7 ± 315.9 ml**	**632.2 ± 263.2 ml**
**MV**	**13.178 ± 6.422 L/min**	**13.278 ± 6.216 L/min**

BMI: Body mass index, RF: respiratory frequency, TV: tidal volume, MV: minute volume

**Table 2:** Comparison with averages and Standard deviation variables: age, BMI, DVC through ventilometry and spirometry in pre and post operatory groups

VARIABLES	PRE OPERATORY AVERAGE ± SD	POST OPERATORY AVERAGE ± SD	*p*
**Age**	**49.7 ± 15.13 years**	**49.7 ± 15.8 years**	**0.985**
**BMI**	**24.8 ± 3.3 kg/m^2^**	**25.5 ± 2.5 kg/m^2^**	**0.353**
**DVCV**	**32.95 ± 11.4 ml/Kg**	**28.27 ± 12.48 ml/Kg**	**0.304**
**DVCI**	**28.9 ± 11 ml/Kg**	**26.98 ± 11 ml/Kg**	**0.859**

BMI: Body mass index, DVCV: direct vital capacity measured with ventilometer. DVCI: direct vital capacity measured with incentive inspirometer.

**Figure 1:**
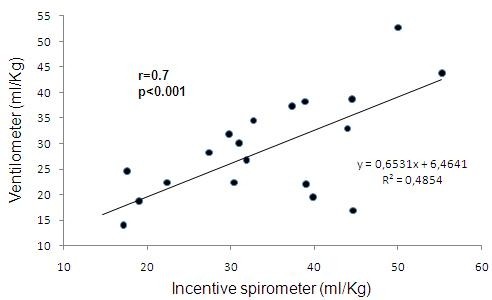
**Correlation between DVC measured with ventilometer and incentive spirometer in pre operatory groups**.

**Figure 2:**
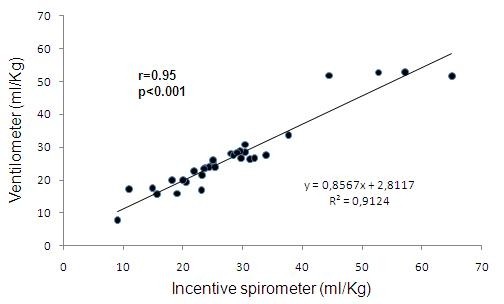
**Correlation between DVC measured with ventilometer and incentive spirometer in post operatory groups**.

Analyzing the obtained data, we can observe that DVC measures between ventilometer and inspirometer show high correlation in pre and post CABG surgery (r = 0.7 and 0.95 respectively, p < 0.01) (Figures 1 and 2).

Figure 1 demonstrates correlation between DVC values in ml/Kg with ventilometer and incentive inspirometer in patients before CABG surgery. DVC with ventilometer varied from 14.6 to 55.2 ml/Kg and DVC with incentive inspirometer varied from 9.2 to 52.7 ml/Kg.

There was a correlation between DVC values in ml/Kg with ventilometer and incentive inspirometer in patients after CABG surgery. DVC with ventilometer varied from 9 to 66.6 ml/Kg and DVC with incentive inspirometer varied from 7.7 to 52.8 ml/Kg (Figure 2).

In subsequent assessment a comparison of averages before and after CABG surgery was performed and it was observed that there were not significant disparities in DVC averages analysis (Table 2).

**Figure 3:**
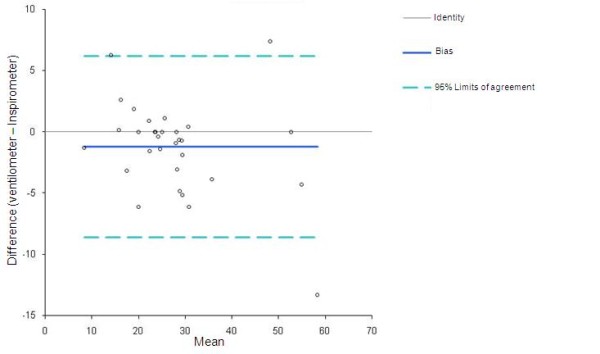
**Bland & Altman plots between Ventilometer and Spirometer pre cardiac surgery**.

**Figure 4:**
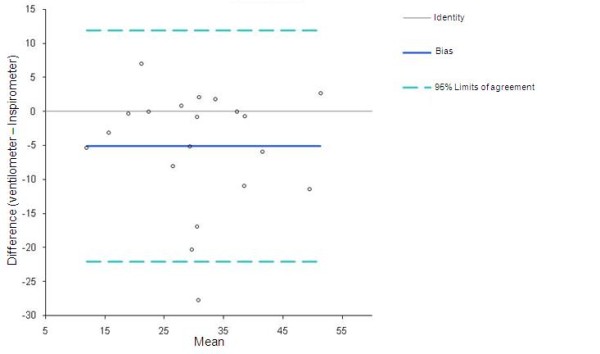
**Bland & Altman plots between Ventilometer and Spirometer post cardiac surgery**.

Bland Altman plots with 95% of agreement are shown in Figures 3 and 4. Cronbach's Alfa index was 0.82 to pre and 0.97 to post surgery.

## Discussion

The main point of this research was the positive correlation between DVC measured with ventilometer and incentive spirometer. Furthermore, the use of incentive spirometer as DVC evaluation method was of easy execution and understanding by the patient.

Nowadays, studies have been carried out with the aim to standardize pulmonary function ways of assessment [26,27]. The investigation of evaluation methods for pulmonary function with high sensibility and specificity has a great value for clinical practice, mainly when these techniques can be applied in a practical way, in bed and at low cost.

In literature there is an array of researches highlighting the importance of volumes and pulmonary capacities measurements. Chevrolet e Deleament [28] assure that VC is an important predictor of pulmonary function because it evaluates the mechanical ventilation necessity and success in ventilatory weaning. Suesada *et al*.[29] showed that VC was one of the variables with higher impairment after short length hospitalization. Gregorini *et al*.[23] reported that patients in post operatory of cardiac surgery showed decreased volumes and pulmonary capacities, therefore reducing the quantity of deep inspirations and cough effectiveness. The lessening of deep inspiration and cough has been proposed to predispose respiratory complications in which atelectasis is the most frequent, reaching approximately 64% of operated patients [23].

The ventilometer, as well as spirometry, is frequently used to evaluate VC in patients with respiratory dysfunctions, however, its cost and maintenance are also elevated [18,19]. This high cost motivates the use of alternative ways to access VC in clinical practice [30].

The rationale of using an incentive spirometer to VC assessment is based on the fact that with ventilometer the patient could perform a deep inspiration until his total pulmonary capacity, followed by continuous and slow expiration until residual volume. With the incentive inspirometer, which operates through inspirations and volumetric registrations, the individual could perform the opposite from the ventilometer and the same air volume would be evaluated. This connection was highly evidenced in the results presented on this research, since the patients in pre and post operatory cardiac surgery showed homogeneous values of DVC measured by ventilometer and incentive spirometer (Figures 1 and 2).

The results of this present study could be used in further investigations in order to deepen the knowledge about the connection between DVC measured with ventilometer and incentive inspirometer.

## Conclusion

There was a high correlation between DVC measures with ventilometer and incentive spirometer in pre and post CABG surgery. Despite this, arises the necessity of further studies to evaluate the repercussion of this method in lowering costs at hospitals.

## Competing interests

The authors declare that they have no competing interests.

## Authors' contributions

VOC and ESRJ were involved with study design, ACP collected the data, MGN and VOC performed the data analysis, MVHR, RAJ and MCMN were involved with data discussion. All authors have read and approved the manuscript.
